# Personality and affective predictors of compensation and implications for intervention recommendations

**DOI:** 10.1002/alz.092739

**Published:** 2025-01-03

**Authors:** Sudev Namboodiri, Sarah Tomaszewski Farias, Danielle J. Harvey, Alyssa Weakley

**Affiliations:** ^1^ University of California, Davis School of Medicine, Sacramento, CA USA

## Abstract

**Background:**

Compensation has the potential to improve everyday functioning and delay or prevent conversion to dementia; thus, improvement of compensation is a desirable intervention target. In addition to cognition, research suggests personality and mood may affect compensation use. The primary aim of this study was to evaluate whether psychological factor(s) are significant predictors of compensation after accounting for cognition in a non‐demented older adult sample. A secondary aim was to examine if there were differences in outcome depending upon whether self or informant report of compensation was included in the model.

**Method:**

Participants included 100 cognitively healthy older adults and 26 individuals with mild cognitive impairment (N = 126; age M = 76.67, SD = 6.79; education M = 15.58, SD = 3.03) who completed the Revised NEO Personality Inventory (used to assess Agreeableness, Neuroticism, Openness, Conscientiousness, and Extraversion) and self‐report measures from the National Institutes of Health (NIH) Toolbox (used to assess Sadness, Self‐efficacy, Anger, and Positive affect). A measurement of Grit was also completed. Self‐ and informant‐report from the Everyday Compensation (EComp) Scale were used as measures of compensation. Linear regression, controlling for cognition (Spanish and English Neuropsychological Assessment Scale Composite) was used to predict compensation (self or informant).

**Result:**

For self‐reported compensation, anger was associated with more compensation independent of cognition (B = .18, t = 2.60, *p* = .01). For informant‐report, Openness was associated with greater compensation (B = .20, t = 2.02, *p* = .05).

**Conclusion:**

Participants with higher scores in Anger and Openness had increased compensation after accounting for cognitive status. A higher degree of Openness indicates a person is willing to try new things, which may result in an increased likelihood of trying compensatory strategies. While it is somewhat counterintuitive that greater negative emotion was associated with more compensation, anger is an activating and externalizing emotion which could facilitate compensation in response to worry about cognitive change. Results suggest that interventions should focus on ways to improve openness and activation to enhance compensation.

